# Health information sharing on social media: quality assessment of short videos about chronic kidney disease

**DOI:** 10.1186/s12882-022-03013-0

**Published:** 2022-11-28

**Authors:** Lan Yao, Yubao Li, Qinglou Lian, Junjun Sun, Shuyin Zhao, Pei Wang

**Affiliations:** 1 Blood Purification Center, the First Affiliated Hospital of Zhengzhou University, 1 East Jianshe Road, Zhengzhou, Henan 450052 China; 2 Research Institute of Nephrology, Zhengzhou University, Zhengzhou, 450052 China; 3 Department of Nephrology, the First Affiliated Hospital of Zhengzhou University, Zhengzhou, 450052 China

**Keywords:** Chronic kidney disease, Health information quality, Infodemiology, Social media, Short video apps

## Abstract

**Introduction:**

Chronic kidney disease (CKD), which affects about 10% of global population, has become a global public health crisis in recent decades. It is well recognized that health information dissemination could change health behaviors, thereby greatly improving the early diagnosis and prevention of diseases. Due to fast dissemination, wide audience, intuitive and vivid, popularization through short videos has rapidly developed into the new main battlefield of health information. The objective of this study was to describe the properties of the CKD-related health information on short video apps.

**Methods:**

Searching on short video apps with high-frequency words in kidney disease as keywords, the basic information of the uploaders was retrieved and extracted short video. Five quality dimensions, awareness, popularity, utility, validity and quality, of each video were assessed with numeric rating scale (NRS) by five volunteers with CKD and three nephrologists.

**Results:**

From the platform of douyin, 65 uploaders and their 3973 short videos of CKD-related health information were investigated in this study. Most information of short videos had relatively high level in awareness, popularity and utility assessment, but some information had relatively low level in validity and quality assessment; 24 (36.9%) uploaders were from governmental hospital (tertiary hospital); 19 uploaders (29.2%) uploaded more than 100 short videos and 49 uploaders (75.4%) updated their videos weekly, and 16 uploaders (24.6%) didn’t update short videos more than one month. There were 4 uploaders (6.2%) have more than 1 million follows, and 39 uploaders (60%) had follows less than 10,000. “Lifestyles”, “Common symptoms of kidney disease” and “Nephritis or kidney disease” were the three main contents of these short videos. The comprehensive data of uploaders with millions of follows in nephrology specialty were much lower than that of orthopedics and other specialty.

**Conclusion:**

The validity and quality of short video is still unsatisfactory, and CKD-related health information also need to be led and improved, although the awareness, popularity, and utility of health information about CKD is acceptable. The public should be selective and cautious in seeking CKD information on social media.

**Supplementary Information:**

The online version contains supplementary material available at 10.1186/s12882-022-03013-0.

## Introduction

Globally, chronic kidney disease (CKD) had become one of the most common chronic noncommunicable diseases, which played a major effect on global health, both as a direct cause of global morbidity and mortality and as an important risk factor for cardiovascular disease [1]. A 2007 survey study showed that the prevalence of CKD in USA rose from 10.0 to 13.1% [2]. A cross-sectional survey from China suggested that 1 in 10 persons living with CKD—indicate nearly 120 million adults suffering from CKD [3]. Typical features of CKD were irreversible, progressive, and strongly associated with higher cardiovascular risk [4]. Unfortunately, most CKD population had no aware of it. For this reason, interventions to prevent and treat CKD, including the dissemination of health information, early screening, and regular physical examinations, were particularly important.

While most people acknowledged personal responsibility for their own health, a survey in the United Kingdom found that 87% of people chose to go to the hospital when they were only “extremely ill” [5]. This had brought a rigorous trial for our doctors and health educators to improve the public society's awareness of medical and health knowledge and the importance of disease [6]. Current research evidence supported the point that health information dissemination could change health behaviors, thereby greatly improving the early diagnosis and prevention of diseases [7]. The diversified development of social media, such as Tik Tok [8–10], Douyin, and Kuaishou, etc., had made the dissemination of public health information develop by leaps and bounds in China and around the world [9, 11, 12]. Similarly, the nephrology community, as one of multiple department of diseases, needs a creative way to popularize health knowledge to improve the world's kidney health problems [13].

Improving the level of education and training of public health personnel was one of the policy priorities according to “Healthy China 2030” blueprint which launched by Chinese government in 2016 [14, 15]. Moreover, popularization of health information was one of the important contents of the work for the association of nephrology. Due to the characteristics of fast dissemination, wide audience, intuitive and vivid, popularization through short videos had rapidly developed into the new main battlefield of health information dissemination. High prevalence and low awareness were the main obstacles to the prevention and treatment of CKD [3, 16–19]. Professional popularization of kidney disease, such as encouraging and correctly guiding social media to carry out high-quality kidney disease health information dissemination under this new situation, would help to improve the public's awareness of CKD, early screening, early diagnosis and prevention strategies for CKD [20–22]. In this study, features of uploaders major in nephrology and their works published on short video apps were investigated and analyzed to explore the current situation of professional popularization of kidney disease on social media in China.

## Research objects and methods

### Research object

Aim of this study is to describe the properties of uploaders (more than 1,000 followers) and their short video works major in kidney disease on Douyin, which is the largest short video social media in China, similar to TikTok.

### Research methods

#### Methods of retrieving research objects

**Fig. 1:**
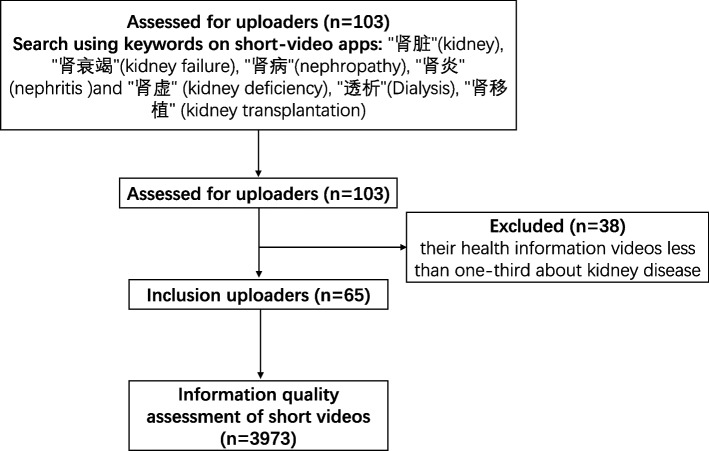
Uploaders and their short videos screening procedure

As described in the previous studies [8, 9], the uploaders and their short videos major in kidney disease were retrieved from Douyin. “kidney”, “kidney failure”, “nephropathy”, “nephritis” and “kidney deficiency”, “dialysis”, “kidney transplantation” and other Chinese high-frequency words used by the public as keywords to search, obtained 103 uploaders with more than 1,000 followers, exclude 38 uploaders whose works about kidney disease less than one-third. A total of 65 uploaders were included in this study (Fig. 1).

Superstar uploaders with followers more than 1 million major in different health information subjects on the Douyin app were retrieved, using Feigua, a data analyzing tool, in August 2020. Dissemination data was compared among these Superstar uploaders.

#### Evaluation methods

Features of these uploaders, such as certification attributes, quantity of works and followers, etc. were evaluated. Quality of their works, which uploaded between April 1^st^ and September 30^th^, 2020, was assessed by three nephrologists and five volunteers with CKD, in whom 2 with diabetes, 2 with primary glomerular disease and 1 with hemodialysis. Five quality dimensions, awareness, popularity, utility, validity and quality, of each works were assessed the with numeric rating scale (NRS) [23, 24], ranging from 0 ≤ very poor < 2, 2 ≤ poor < 4, 4 ≤ fair < 6, 6 ≤ good < 8, 8 ≤ excellent ≤ 10, and the average opinion score (Mean Opinion Score, MOS) was taken.

### Ethical requirements

This study used publicly available data and anonymized analysis, institutional review board review and informed consent requirements were waived.

### Statistical analysis

Database was established with Microsoft Excel and implement two-person verification data entry. Descriptive statistical analysis was performed on counting data.

## Result

### Quality of short video works

Quality of 3973 short videos were evaluated by CKD volunteers and nephrologists, Table 1. The “awareness” of most short videos was 0–4 points (78.1%), the “popularity” was focused on 6–8 points (57.1%), and the “utility” was focused on 6–8 points (56.5). The “validity” of most short videos focused on 6–10 points (75.4%), and the “quality” was mainly scattered in 4–10 points.

**Table 1:** Scoring results of 3973 short videos of health information about kidney disease, n (%)

	I	II	III	IV	V
Awareness	19 (0.5)	337 (8.5)	909 (22.9)	1780 (44.8)	928 (23.3)
Popularity	538 (13.5)	2268 (57.1)	741 (18.7)	299 (7.5)	127 (3.2)
Utility	638 (16.1)	2243 (56.5)	759 (19.1)	200 (5.0)	133 (3.3)
Validity	1753 (44.1)	1243 (31.3)	622 (15.7)	287 (7.2)	68 (1.7)
Quality	1051 (26.4)	1437 (36.2)	1006 (25.3)	392 (9.9)	87 (2.2)

8 points < I ≤ 10 points, 6 points < II ≤ 8 points, 4 points < III ≤ 6 points, 2 points < IV ≤ 4 points, 0 points ≤ V ≤ 2 points

### The disease classification of short video works

A total of 3973 short video works were classified according to their topics, which including the following 13 types, common symptoms of kidney disease, nephritis or kidney disease, diabetic kidney disease, or hypertension, and so on. Lifestyles related works accounted for the highest proportion (21.3%). Followed by “Common symptoms of kidney disease” (19.3%), including frequent urination and abnormal urine test, edema, proteinuria, and obesity. The third was “Nephritis or kidney disease”, accounting for 14%, Table 2.

**Table 2:** Disease classification of 3973 short videos content

Disease classification	Videos, n(%)
**Common symptoms of kidney disease**	769 (19.3)
Frequent urination and abnormal urine test	554 (13.9)
Edema	25 (0.6)
Proteinuria	168 (4.3)
Obesity	22 (0.5)
**Nephritis or kidney disease**	562 (14.2)
IgA	92 (2.3)
Nephrotic syndrome	75 (1.9)
Nephritis	42 (1.1)
Unspecified or generalized kidney disease	353 (8.9)
**Diabetic kidney disease**	151 (3.8)
**Hypertension**	67 (1.7)
**Chronic kidney disease or kidney failure**	235 (5.9)
**Hereditary kidney disease**	16 (0.4)
**Kidney stones**	50 (1.3)
**Hyperuricemia**	310 (7.8)
**TCM nephropathy**	134 (3.4)
**Drug-induced kidney injury or medication**	227 (5.7)
**Lifestyles**	848 (21.3)
**Diet and drinking**	285 (7.2)
**Others**	319 (8.0)

### The certification attributes of the uploaders on social media

**Fig. 2:**
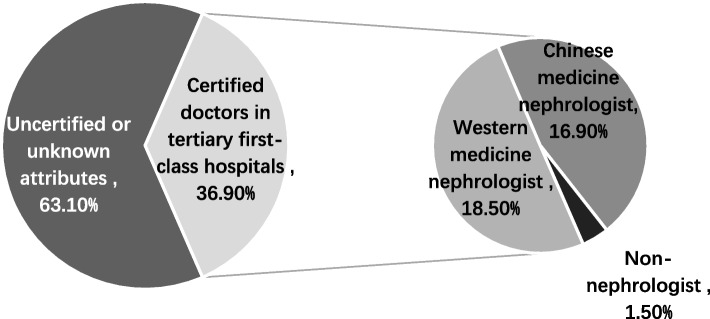
Authentication attribute distribution of the CKD-related uploaders on short videos apps

Among the 65 uploaders of health information dissemination about renal specialty, 24 (36.9%) had yellow-V certification (verified of the medical profession) or worked in a tertiary A hospital, of which 12 were Western medicine nephrologists, 11 were traditional Chinese medicine (TCM) nephrologists, 1 was a non-nephrologist. The other (63.1%) were not certified or have unknown attributes (Fig. 2).

### Features of uploaders

**Fig. 3:**
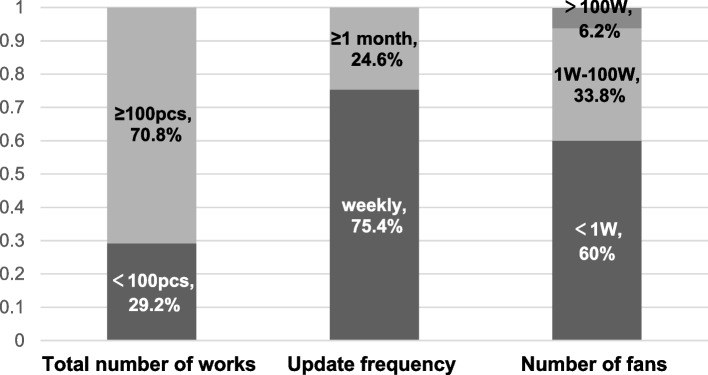
Analysis the basic dada of the short videos published by the uploaders of health information about kidney disease in the past six months

On Douyin app, 19 uploaders (29.2%) uploaded more than 100 works, other 46 uploaders (70.8%) uploaded less than 100 works in the six months survey period. In addition, 16 uploaders (24.6%) did not update their short video works in the past month, the other 49 uploaders (75.4%) updated their works at least weekly. Only 4 uploaders (6.2%) had more than 1 million follows, and most uploaders (39, 60%) had less than 10,000 follows, 22 (33.8%) uploaders had follows between 10,000 and 1 million, Fig. 3.

### Comparison of nephrology superstars with other majors

Five superstars majored nephrology, with total 12,709 million follows and 725 short videos, and the highest number of “likes” for a single work is 1,192 million. Compared with ones major in other fields, data of superstars major in nephrology had different degrees of advantages and disadvantages, as shown in Table 3. Data were lower than the superstars of orthopedics, obstetrics, and gynecology, but each has own advantages compared with who major in urology, cardiology, dermatology, and pediatrics.

**Table 3:** Comparison of renal professional superstars of health information dissemination on short video apps with other professions

Professional	Quantity (unit: piece)	Total number of follows (ten thousand)	Total number of works (pieces)	The highest number of “likes” for a single work (ten thousand)
Nephrology	5	1270.9	725	119.2
Orthopedics	13	4277.5	2054	114.1
Obstetrics and Gynecology	8	2344.1	1216	154.8
Urology	5	950.8	895	131.5
Cardiology	4	1358.7	1731	30.6
Dermatology	5	3175.4	706	194.2
Pediatrics	5	1175.8	664	182.9

## Discussion

The popularization played an important role in promoting healthy lifestyles and disease information dissemination [25], which could improve the public’s awareness of diseases and the overall level of prevention and treatment of disease in China. Popularization of health information had been included in the construction goals and related work plans of the "Healthy China 2030" Planning Outline [26]. Recently, health information dissemination was affected by the pandemic of COVID-19 greatly [27]. Due to the characteristics of strong dissemination and acceptance, Internet especially social media platforms, such as short video apps, had gradually become one of the main fields of health information dissemination [28, 29], and played an increasingly important role in sub-healthy or potentially diseased populations [30, 31].

The construction of the prevention and treatment system for CKD was inseparable from health information dissemination. Through popularization of health information, strengthening the public's awareness and attention to CKD, early detection and diagnosis of CKD, and understanding of CKD prevention and treatment strategies were extremely important for CKD management [32–34]. The CKD prevention and control work plan in Taiwan, China since 2002 had been carried out through more than ten years, which had significantly reduced the incidence of end-stage renal disease (ESRD) [21]. Therefore, it had significant academic and social value to encourage and carry out health information popularization of kidney disease [35–37]. At present, short videos popularization of health information about kidney disease was in the ascendant. This study investigated the status of short video type of health information dissemination on social media platforms and provided a basis for the society to encourage and formulate a health information popularization work.

Currently, uploaders who share health information on Douyin app were demanded to provide certification materials for working in tertiary A hospitals for doctor’s identification. This study found that most uploaders (63.1%) did not have doctor identification, indicating that nephrologists in tertiary A hospitals with high professional level lacked enthusiastic to participate health information popularization of kidney disease. It also showed that the quality of some short video works needed to be improved in the survey. More than half of the uploaders uploaded their first work within 6 months during the survey, and most of them started to carry out popularization of health information work during the COVID-19 pandemic. This was in line with the drastic increase in public demand for health knowledge in crisis situations. Nearly a quarter of the uploaders had not updated their works more than a month, indicating that there was a high probability that they would withdraw from this work. 60% of the uploaders had less than 10,000 follows, and their influence was relatively weak. The results of this survey showed that kidney disease information popularization of short video on social media emerged spontaneously and was still in the preliminary stage. More senior nephrologist should be encouraged to participate in kidney disease popularization. Special training and promotion should be carried out to meet the public's demand for renal health knowledge.

Awareness, popularity, utility, validity, and quality are the five main elements of health information dissemination. CKD volunteers and nephrologists were invited to evaluate the quality of these works. Most works had low scores for “awareness” and high scores for “utility”, indicating that the topic selection of most health information was appropriate and helpful for improving the public’s information of kidney disease. It also showed that the development of health information popularization satisfied public’s demands. It was also found that most of the videos had relatively high scores for “popularity”, indicating that short video works were indeed a form of medical popularization that spreads quickly and was popular for publics. However, in the scoring of the professionalism of nephrologists, the “validity” and “quality” scores of some works were relatively low, indicating that quality of these works was uneven, and some works were even misleading to a certain extent. The results of this research showed that health information popularization still needed to be properly guided, so that high-quality ones occupied the main battlefield.

The limitations of this study were as follows: First, only the uploaders in Douyin platform were analyzed, those uploaded popularization works in other short video platforms were not included. In addition, language or ethnic differences might produce different results since this study only assessed the health information quality of Chinese short videos. Last but not least, quality of works was assessed by five CKD volunteers and three nephrologists, and bias might exist for their own different knowledge. In future research, we will call for more extensive cross-language comparative studies across different social media apps using more precise assessment tools.

## Conclusion

This study assessed features of 65 kidney disease popularization uploaders and quality of their 3973 short video works on Douyin app in China. The results showed that most uploaders did not obtain doctor’s identification. Awareness, popularity, and utility of these works was satisfactory, although validity and quality varied across different uploaders. The results suggested that more nephrologists should be encouraged to participate in kidney disease popularization and the uploaders should be specificized.

## Supplementary Information

**Additional file 1.**

## Data Availability

All data during the study appear in the submitted article/supplementary material; further inquiries are available from the corresponding author by request.

## Abbreviations

CKD: Chronic kidney disease
ESRD: End-stage renal disease

## References

[1] Global, regional, and national burden of chronic kidney disease, 1990–2017: a systematic analysis for the Global Burden of Disease Study 2017. Lancet. 2020;395:709–733. 10.1016/s0140-6736(20)30045-3. 10.1016/S0140-6736(20)30045-3 PMC7049905 32061315

[2] Coresh J (2007). Prevalence of chronic kidney disease in the United States. JAMA.

[3] Zhang L (2012). Prevalence of chronic kidney disease in China: a cross-sectional survey. Lancet.

[4] Lv JC, Zhang LX (2019). Prevalence and disease burden of chronic kidney disease. Adv Exp Med Biol.

[5] McCarthy K, Prentice P (2006). Commissioning health education in primary care. BMJ.

[6] Education for health (1990). A role for physicians and the efficacy of health education efforts. Council on scientific affairs. JAMA.

[7] Allen K, McFarland M (2020). How are income and education related to the prevention and management of diabetes?. J Aging Health.

[8] Song S (2021). Short-video apps as a health information source for chronic obstructive pulmonary disease: information quality assessment of TikTok videos. J Med Internet Res.

[9] Kong W, Song S, Zhao YC, Zhu Q, Sha L (2021). TikTok as a health information source: assessment of the quality of information in diabetes-related videos. J Med Internet Res.

[10] Wu H (2022). The current state of vascular surgery presence in Bilibili video platform of China. Front Surg.

[11] Wu T, Li L (2017). Evolution of public health education in China. Am J Public Health.

[12] Bautista JR, Zhang Y, Gwizdka J (2021). Healthcare professionals' acts of correcting health misinformation on social media. Int J Med Informatics.

[13] Brewster UC (2021). Global health education: a call to action in this moment. Am J Kidney Dis.

[14] Advancing China's health and medical science. Lancet. 2015;386:1796. 10.1016/s0140-6736(15)00811-9. 10.1016/S0140-6736(15)00811-9 26843292

[15] Kleinert S, Horton R (2014). How should medical science change?. Lancet.

[16] Provenzano M (2019). Epidemiology of cardiovascular risk in chronic kidney disease patients: the real silent killer. Rev Cardiovasc Med.

[17] Ene-Iordache B (2016). Chronic kidney disease and cardiovascular risk in six regions of the world (ISN-KDDC): a cross-sectional study. Lancet Glob Health.

[18] Kumela Goro K (2019). Patient awareness, prevalence, and risk factors of chronic kidney disease among diabetes mellitus and hypertensive patients at Jimma University Medical Center, Ethiopia. BioMed Res Int.

[19] Wen CP (2008). All-cause mortality attributable to chronic kidney disease: a prospective cohort study based on 462 293 adults in Taiwan. Lancet.

[20] Wei SY (2010). Chronic kidney disease care program improves quality of pre-end-stage renal disease care and reduces medical costs. Nephrology (Carlton).

[21] Chen YR (2014). Multidisciplinary care improves clinical outcome and reduces medical costs for pre-end-stage renal disease in Taiwan. Nephrology (Carlton).

[22] Chen PM (2015). Multidisciplinary care program for advanced chronic kidney disease: reduces renal replacement and medical costs. Am J Med.

[23] Kimel M, Zeidler C, Kwon P, Revicki D, Ständer S (2020). Validation of psychometric properties of the itch numeric rating scale for pruritus associated with Prurigo Nodularis: a secondary analysis of a randomized clinical trial. JAMA Dermatol.

[24] Chiarotto A (2019). Measurement properties of visual analogue scale, numeric rating scale, and pain severity subscale of the brief pain inventory in patients with low back pain: a systematic review. J Pain.

[25] Hunziker S (2022). Effect of bedside compared with outside the room patient case presentation on patients' knowledge about their medical care. Ann Intern Med.

[26] Chen P, Li F, Harmer P (2019). Healthy China 2030: moving from blueprint to action with a new focus on public health. Lancet Public health.

[27] Kaul V (2021). Medical education during the COVID-19 pandemic. Chest.

[28] van der Keylen P (2020). The online health information needs of family physicians: systematic review of qualitative and quantitative studies. J Med Internet Res.

[29] Goobie GC, Guler SA, Johannson KA, Fisher JH, Ryerson CJ (2019). YouTube videos as a source of misinformation on idiopathic pulmonary fibrosis. Ann Am Thorac Soc.

[30] Diaz JA (2002). Patients' use of the Internet for medical information. J Gen Intern Med.

[31] Grosberg D, Grinvald H, Reuveni H, Magnezi R (2016). Frequent surfing on social health networks is associated with increased knowledge and patient health activation. J Med Internet Res.

[32] Levey AS (2020). Nomenclature for kidney function and disease: report of a Kidney Disease: Improving Global Outcomes (KDIGO) consensus conference. Kidney Int.

[33] Chen TK, Knicely DH, Grams ME (2019). Chronic kidney disease diagnosis and management: a review. JAMA.

[34] Kalantar-Zadeh K, Jafar TH, Nitsch D, Neuen BL, Perkovic V (2021). Chronic kidney disease. Lancet.

[35] Ebrahimi H, Sadeghi M, Amanpour F, Dadgari A (2016). Influence of nutritional education on hemodialysis patients' knowledge and quality of life. Saudi J Kidney Dis Transpl.

[36] Jha V (2013). Chronic kidney disease: global dimension and perspectives. Lancet.

[37] Siew ED (2019). Kidney disease awareness and knowledge among survivors ofacute kidney injury. Am J Nephrol.

